# Bacterial vaginosis and adverse outcomes among full-term infants: a cohort study

**DOI:** 10.1186/s12884-016-1073-y

**Published:** 2016-09-22

**Authors:** Adam S. Dingens, Tessa S. Fairfortune, Susan Reed, Caroline Mitchell

**Affiliations:** 1 Department of Epidemiology, University of Washington School of Public Health, 1959 NE Pacific Street Health Sciences Bldg, Seattle, WA 98195 USA; 2 University of Washington, Molecular & Cellular Biology Program, 1959 NE Pacific St, Seattle, WA 98195 USA; 3 Fred Hutchinson Cancer Research Center, 1100 Fairview Ave N, Seattle, WA 98109 USA; 4 Harborview Medical Center, 325 Ninth Ave, Seattle, WA 98104 USA; 5 Vincent Center for Reproductive Biology, Massachusetts General Hospital, 55 Fruit Street, Boston, MA 02114 USA; 6 Harvard Medical School, 25 Shattuck St, Boston, MA 02115 USA

**Keywords:** Bacterial vaginosis, Term, Adverse neonatal outcomes, Neonatal sepsis

## Abstract

**Background:**

Bacterial vaginosis (BV) during pregnancy is a well-established risk factor for preterm birth and other preterm pregnancy complications. Little is known about adverse neonatal outcomes associated with BV exposure in full-term births, nor its influence on adverse outcomes independent of its effect on gestational age. The purpose of this study was to examine the relationship between BV during pregnancy and adverse neonatal outcomes among full-term and preterm infants.

**Methods:**

We conducted a retrospective cohort study of Washington State mother/infant pairs from 2003-2013, stratified by full-term (primary outcomes) and preterm births (secondary outcomes). BV-exposed and unexposed women were frequency-matched based on year of delivery. BV exposure and adverse outcomes [assisted ventilation/respiratory distress, neonatal intensive care unit (NICU) admission, neonatal sepsis, fetal mortality, and infant mortality] were identified using birth certificates, ICD-9 codes from linked hospital records, and death certificates. Associations between BV exposure and outcomes were assessed using multivariable Poisson regression, adjusted for maternal demographics, gestational age, and other pregnancy complications, including infections.

**Results:**

A total of 12,340 mother/infant pairs were included: 2,468 BV-exposed (2198 term, 267 preterm) and 9,872 BV unexposed (9156 term, 708 preterm). Among full-term infants, BV-exposed mothers were younger, more likely to be Black or Hispanic, more likely to have had a sexually transmitted infection, and less likely to have a college degree than unexposed mothers. Term BV exposed infants were more likely to have meconium at delivery. Following adjustment, BV was associated with an increased risk of assisted ventilation/respiratory distress at birth (aRR = 1.28, 95 % CI 1.02-1.61), NICU admission (aRR = 1.42, 95 % CI 1.11-1.82), and neonatal sepsis (aRR = 1.60, 95 % CI 1.13-2.27) among full-term infants. These associations were independent of the presence of chorioamnionitis or meconium. Among preterm infants, BV-exposure was associated with an increased risk for NICU admissions only (aRR = 1.24, 95 % CI 1.04-1.46).

**Conclusions:**

BV exposure during pregnancy is associated with adverse neonatal outcomes even among infants born full-term. These findings amongst full-term infants are novel, and highlight neonatal implications of BV in pregnancy independent of BV’s effect on preterm birth.

## Background

Bacterial vaginosis (BV), defined as a disturbance in the natural vaginal microbiota characterized by reduced *Lactobacillus* and overgrowth of anaerobic bacteria [1], is found in 9 % to 23 % of pregnant women [2]. Symptoms include vaginal discharge and malodor, but often women who have BV are asymptomatic. BV during pregnancy is an established risk factor for preterm delivery [1, 3–5] as well as other adverse preterm pregnancy outcomes such as premature rupture of membranes, low birth weight, chorioamnionitis, and spontaneous abortion [1, 4, 5]. A single cohort study of preterm infants suggests that BV is associated with an increased risk of adverse neonatal outcomes including: NICU admission, respiratory distress at birth, requirement of assisted ventilation at birth, and NICU stays lasting longer than 2 days [6]. Data on adverse neonatal outcomes associated with BV among infants born full-term is almost nonexistent.

We hypothesized that BV is associated with an increase in adverse neonatal outcomes due to ascending infection *at all gestational ages* and that the increased risks previously observed among preterm pregnancies are not solely attributable to prematurity. The primary aim of this large Washington State cohort study was to evaluate, *among full-term infants*, the effects of BV on five fetal/infant outcomes: assisted ventilation/respiratory distress at birth, admission to neonatal intensive care unit (NICU), neonatal sepsis, fetal mortality, and infant mortality. Our secondary aims were to evaluate these same outcomes among preterm (<37 weeks gestation) infants exposed and unexposed to BV, as well as to reaffirm the relationship between BV and preterm delivery among all infants. Women included as BV-exposed in our study were likely symptomatic women, and asymptomatic women were likely disproportionately misclassified in the unexposed group. However, this misclassification would only drive findings toward the null - any association found in our study would be perhaps, “only the tip of the iceberg”, or a marker for an important underlying etiology related to BV but not directly causal.

## Methods

We conducted a population-based retrospective cohort study using data from Washington State birth certificates and birth hospitalizations from 2003-2013. These data were extracted from the Birth Events Records Database (BERD), a database linking more than 95 % of Washington State birth certificate data with maternal and infant hospitalization records from the statewide Comprehensive Hospital Abstract Report System (CHARS) [7]. Infant mortality was identified from death certificates issued through 2014. While there is underreporting of birth complications and maternal conditions on birth certificates [8], the use of hospital discharge and death certificate data improves the accuracy and completeness of both exposures and outcomes in this study. Four of our 5 outcomes: assisted ventilation/respiratory distress at birth, admission to neonatal intensive care unit (NICU), fetal mortality, and infant mortality are “hard outcomes” and it is highly unlikely that they would be inaccurate. Neonatal sepsis, our fifth outcome, is perhaps more prone to inaccurate birth certificate reporting [8], but it is also likely to be captured in linked hospital discharge data, as the sensitivity of ascertaining infection-related outcomes using ICD9 codes is near perfect [9]. All data were de-identified, and the study was determined to be exempt from review by the Washington State Institutional Review Board.

### Study population

We included all BV-exposed mothers who gave birth in Washington State from 2003-2013 who met study criteria. Women were considered BV-exposed if on the birth certificate the “other” box under “Infections Present and/or Treated During this Pregnancy” was checked and the write-in space specified the infection as bacterial vaginosis (e.g., “BV”, “Bac Vag”, or “B Vaginosis”). Additional exposed women were identified using ICD-9 codes 616.10, 616.11, and 646.63 from CHARS data. BV-unexposed women did not have a write-in infection that could be identified as BV nor any BV ICD-9 codes in their delivery hospitalization records. We enrolled four unexposed women per exposed woman, frequency-matched for the infant’s year of birth. Non-singleton and births with a congenitally malformed infant were excluded.

### Primary outcome measures

We measured the risk of five fetal/infant outcomes: assisted ventilation/respiratory distress at birth, NICU admission, neonatal sepsis, fetal mortality, and infant mortality. Outcomes were identified using a combination of birth certificate checkboxes, ICD-9 codes from the birth hospitalization record, and death certificates. If either the box on the birth certificate was checked, or the ICD-9 code was present, the outcome was considered to have occurred. Assisted ventilation/respiratory distress is coded on the birth certificate as “assisted ventilation required immediately following birth” and was also evaluated using ICD-9 codes 768.5 (severe birth asphyxia) and 769 (respiratory distress syndrome in a newborn). NICU admission was identified via birth certificate checkbox. Neonatal sepsis was ascertained using ICD-9 codes 771.81 (septicemia [sepsis] of newborn) and 771.83 (bacteremia of newborn) and by birth certificate checkbox. Because this study primarily used birth certificate data, fetal deaths before 20 weeks gestation (where no birth or death certificate would have been issued) were not captured. Fetal mortality at 20 weeks or greater gestation was identified from birth hospitalization records, ICD-9 code V27.1, and linked death certificates. Infant mortality is defined as death within one year of birth, and was identified via linked birth and death certificates.

### Statistical analyses

We used Poisson regression to calculate risk ratios with 95 % confidence intervals for all outcomes among sub-cohorts of full-term infants (≥37 weeks gestation) and preterm infants (<37 weeks gestation), as well as the complete cohort. Gestational age was established from the birth certificate, primarily from ultrasound dating in the first or second trimester (*N* = 12,317); when this variable was missing, we estimated gestational age using the date of the mother’s last menstrual period (*N* = 23). We established *a priori* that maternal age and maternal race (White, Black, Hispanic, Asian, Native American, Other) would be included in the models [3, 5, 10, 11]. We also controlled for gestational age when appropriate.

In addition, we considered the following as covariates: maternal education (<high school, high school graduate/some college, college degree); delivery method (cesarean delivery or vaginal birth, either spontaneous or assisted); prenatal smoking (yes/no); nulliparity (yes/no); birth weight <2500 grams (yes/no); small for gestational age among live born infants (lowest 10th percentile, calculated from the BERD database from 1989-2002 based on Lipsky *et al*.) [12]; sexually transmitted infection (STI; syphilis, gonorrhea, chlamydia, or genital herpes) diagnosed and/or treated during pregnancy (yes/no); presence of hypertension, diabetes, or pre-eclampsia during pregnancy (yes/no); presence of chorioamnionitis (yes/no); and presence of moderate or heavy meconium (yes/no). Covariates with significant missing data were not included in the models. A variable was considered to be a potential confounder if controlling for the variable produced a >10 % change in the risk estimate. Confounding was assessed separately in the full-term and preterm sub-cohorts, as well in the entire cohort for all outcomes. Confounders determined not to be in the causal pathway were included in the model. Significant associations between BV and the examined outcomes were further explored with stratified analyses evaluating our hypothesis that poor outcomes were likely to be associated with an ascending infection.

All analyses were conducted using STATA 13 software (Stata Corporation, College Station, Texas).

## Results

We included and analyzed 12,340 mother/infant pairs. Gestational age was missing for 11 infants; these participants were excluded from the full-term (*N* = 11,354) and pre-term (*N* = 975) sub-cohort analyses but were included for analyses of the full cohort. There were 2,198 term and 267 preterm mother/infant pairs identified that were BV exposed.

Demographic characteristics varied between BV exposed and unexposed women (Table 1). Among full-term birth, BV-exposed mother were significantly younger than unexposed mothers (<20 years: 14.7 % vs. 7.3 %), and more likely to be having their first birth (47.2 % vs. 41.4 %). They were more likely to be Black (10.7 % vs. 4.6 %) or Hispanic (19.0 % vs. 10.8 %), and less likely to have a college degree (16.5 % vs. 36.1 %). BV-exposed mothers were more likely to have smoked during pregnancy (16.5 % vs. 10.1 %), less likely to have had a cesarean delivery (21.7 % vs. 27.1 %), more likely to have a small for gestational age infant (10.3 % vs. 8.6 %), less likely to have had at least adequate prenatal care as indicated by the Kotelchuck index (59.6 % vs. 62.5 %), and more likely to have had an STI diagnosed and/or treated during their pregnancy (13.5 % vs. 4.1 %). Comorbid pregnancy conditions (diabetes, hypertension and preeclampsia) were similar between BV-exposed and BV-unexposed mothers. Term infants born to BV-exposed mothers were more likely to have moderate or heavy meconium (9.0 % vs. 5.6 %) than infants of unexposed mothers. Preterm infants born to BV-exposed mothers had a higher risk of chorioamnionitis (8.2 % vs. 5.8 %). Other demographic characteristics were similar in the full-term and preterm births, with the exception that cesarean deliveries and low birth weight infants were higher in the preterm births.

**Table 1:** Maternal characteristics of women with and without bacterial vaginosis during pregnancy, Washington State 2003-2013

	Full Term (*N* = 11,354)				Preterm (*N* = 975)				Total (*N* = 12,340)			
	BV exposed		BV unexposed		BV exposed		BV unexposed		BV exposed		BV unexposed	
	*N* = 2198		*N* = 9156		*N* = 267		*N* = 708		*N* = 2468		*N* = 9872	
	*n*	*%*	*n*	*%*	*n*	*%*	*n*	*%*	*n*	*%*	*n*	*%*
Age (years)*												
<20	324	*14.7*	666	*7.3*	39	*14.6*	56	*7.9*	363	*14.7*	723	*7.3*
20-24	784	*35.7*	2095	*22.9*	79	*29.6*	179	*25.3*	866	*35.1*	2278	*23.1*
25-29	620	*28.2*	2593	*28.3*	66	*24.7*	166	*23.5*	686	*27.8*	2760	*28.0*
30-34	317	*14.4*	2375	*26.0*	44	*16.5*	188	*26.6*	361	*14.6*	2564	*26.0*
35+	153	*7.0*	1424	*15.6*	39	*14.6*	119	*16.8*	192	*7.8*	1544	*15.6*
Race/Ethnicity*												
White	1268	*58.8*	6842	*71.7*	152	*58.9*	497	*71.8*	1422	*58.8*	6981	*71.7*
Black	231	*10.7*	416	*4.6*	21	*8.1*	36	*5.2*	252	*10.4*	452	*4.6*
Asian	183	*8.5*	939	*10.4*	28	*10.9*	64	*9.3*	212	*8.8*	1006	*10.3*
Hispanic	410	*19.0*	979	*10.8*	39	*15.1*	73	*10.6*	449	*18.6*	1054	*10.8*
Native American	62	*2.9*	218	*2.4*	18	*7.0*	22	*3.2*	80	*3.3*	240	*2.5*
Other	4	*0.2*	9	*0.1*	0	*0.0*	0	*0.0*	4	*0.2*	9	*0.1*
Education*												
Less than H.S.	689	*31.6*	1639	*18.1*	89	*34.1*	148	*21.4*	779	*31.9*	1791	*18.4*
H.S. grad/some college	1132	*51.9*	4134	*45.8*	129	*49.4*	323	*46.6*	1263	*51.7*	4460	*45.8*
College graduate	360	*16.5*	3263	*36.1*	43	*16.5*	222	*32.0*	403	*16.5*	3486	*35.8*
Nulliparous*	3733	*47.2*	1035	*41.4*	118	*45.2*	309	*45.2*	1154	*47.0*	4045	*41.7*
Cesarean delivery*	477	*21.7*	2479	*27.1*	78	*29.3*	269	*38.1*	555	*22.5*	2751	*27.9*
Low Birth Weight (<2500 g)*	41	*1.9*	139	*1.5*	158	*60.1*	344	*49.1*	199	*8.1*	483	*4.9*
Small for gestational age	227	*10.3*	790	*8.6*	22	*8.2*	76	*10.7*	249	10.1	866	*8.8*
missing	0	*(0)*	12	*(0.13)*	20	*(7.49)*	15	*(2.12)*	23	(0.93)	35	*(0.35)*
Smoking*	360	*16.5*	921	*10.1*	42	*15.9*	84	*12.0*	404	*16.5*	1006	*10.3*
Kotelchuck Index												
Inadequate	374	*17.8*	1203	*15.2*	29	*14.2*	85	*16.7*	403	*17.5*	1291	*15.3*
Intermediate	475	*22.6*	1763	*22.3*	39	*19.1*	76	*14.9*	514	*22.3*	1839	*21.8*
Adequate	899	*42.7*	3658	*46.3*	52	*25.5*	150	*29.5*	951	*41.2*	3808	*45.2*
Adequate Plus	356	*16.9*	1283	*16.2*	84	*41.2*	198	*38.9*	440	*19.1*	1481	*17.6*
missing	94	*(4.3)*	1249	*(13.6)*	63	*(23.6)*	199	*(28.1)*	160	*(6.5)*	1453	*(14.7)*
STI*†	296	*13.5*	379	*4.1*	34	*12.7*	42	*5.9*	330	*13.4*	422	*4.3*
Maternal comorbidities‡	236	*10.8*	1022	*11.3*	53	*20.0*	184	*26.3*	289	*11.7*	1207	*12.4*
missing	2	*(0.1)*	92	*(1.0)*	2	*(0.8)*	7	*(1.0)*	5	*(0.2)*	99	*(1.0)*
Chorioamnionitis*	62	*2.8*	265	*2.9*	22	*8.2*	41	*5.8*	84	*3.4*	306	*3.1*
Meconium (moderate/heavy)	198	*9.0*	510	*5.6*	6	*2.3*	19	*2.7*	204	*8.3*	530	*5.4*
missing	4	*(0.2)*	98	*(1.1)*	20	*(7.5)*	26	*(3.7)*	25	*(1.0)*	126	*(1.3)*

*BV* bacterial vaginosis; *H.S*. High School, *STI* sexually transmitted infections

*Missing data not displayed, <3.5 % in all groups

†syphilis, gonorrhea, chlamydia, and/or genital herpes

‡hypertension, diabetes, and/or pre-eclampsia

Among full-term infants, BV exposure was significantly and positively associated with an approximately 28 % increased risk of assisted ventilation at birth, a 42 % increased risk for admission to NICU and a 60 % increased risk for neonatal sepsis after adjusting for gestational age, maternal age, and maternal race (Table 2). There was no association with fetal or infant mortality. The presence of an STI confounded the relationship between BV and fetal mortality in the full-term births and was controlled for, but presence of an STI did not confound any other outcomes in term, preterm, or all births. Additionally, no other covariates confounded the relationship between BV and any of the outcomes examined. Among full-term births, the risk ratio for the association of BV with neonatal sepsis was higher in SGA infants (adjusted risk ratio [aRR] = 2.57, 95 % confidence interval [CI] 1.04, 6.40) than in AGA and LGA infants (aRR = 1.46, 95 % CI 1.00, 2.13), but confidence intervals are overlapping. Rates of chorioamnionitis and meconium were higher in BV-exposed pregnancies, but neither modified the relationship between BV and the outcomes examined (Table 3).

**Table 2:** Associations between bacterial vaginosis and adverse outcomes among full-term infants (37 weeks or greater gestation), Washington State, 2003-2013, adjusted for gestational age, maternal age, and maternal race (*N* = 11,354)

	BV exposed		BV unexposed		RR	95 % CI	aRR	95 % CI
	*N* = 2198		*N* = 9156					
	*n*	*%*	*n*	*%*				
Assisted ventilation at birth	100	*4.6*	331	*3.6*	1.26	1.01–1.57	1.28	1.02–1.61
Admission to NICU at birth	89	*4.1*	272	*3.0*	1.36	1.08–1.72	1.42	1.11–1.82
Neonatal sepsis	48	*2.2*	126	*1.4*	1.59	1.14–2.21	1.60	1.13–2.27
Fetal Mortality	3	*0.1*	13	*0.1*	0.96	0.27–3.37	1.15*	0.27–4.96
Infant mortality	4	*0.2*	24	*0.3*	0.69	0.24–2.00	0.39	0.12–1.28

*BV* bacterial vaginosis, *RR* risk ratio, *aRR* adjusted risk ratio, *CI* confidence interval, *NICU* neonatal intensive care unit

*further adjusted for presence of sexually transmitted infections (syphilis, gonorrhea, chlamydia, and/or genital herpes)

**Table 3:** Associations between bacterial vaginosis and adverse outcomes among full-term infants (37 weeks or greater gestation), Washington State, 2003-2013 (*N* = 11,354), adjusted for gestational age, maternal age, and maternal race, and additionally adjusted for the presence of chorioamnionitis or meconium

	BV exposed		BV unexposed		aRR	95 % CI
	*N* = 2198		*N* = 9156			
	*n*	*%*	*n*	*%*		
Assisted ventilation at birth	98	*4.5*	328	*3.6*	1.28	1.02–1.61
additionally adjusted for chorioamnionitis	98	*4.5*	328	*3.6*	1.28	1.02–1.61
additionally adjusted for meconium	98	*4.5*	328	*3.7*	1.22	0.97–1.53
Admission to NICU at birth	88	*4.1*	269	*3.0*	1.42	1.11–1.82
additionally adjusted for chorioamnionitis	88	*4.1*	269	*3.0*	1.45	1.13–1.85
additionally adjusted for meconium	88	*4.1*	267	*3.0*	1.34	1.05–1.72
Neonatal sepsis	48	*2.2*	125	*1.4*	1.60	1.13–2.27
additionally adjusted for chorioamnionitis	48	*2.2*	125	*1.4*	1.67	1.19–2.35
additionally adjusted for meconium	48	*2.2*	125	*1.4*	1.51	1.06–2.14
Fetal Mortality	3	*0.1*	12	*0.1*	1.15*	0.27–4.96
additionally adjusted for chorioamnionitis	3	*0.1*	12	*0.1*	1.14*	0.27–4.88
additionally adjusted for meconium	0	*0.0*	3	*0.0*	-	-
Infant mortality	3	*0.1*	24	*0.3*	0.39	0.12–1.28
additionally adjusted for chorioamnionitis	3	*0.1*	24	*0.3*	0.39	0.12–1.27
additionally adjusted for meconium	3	*0.1*	23	*0.3*	0.40	0.12–1.34

*BV* bacterial vaginosis, *aRR* adjusted risk ratio, *CI* confidence interval, *NICU* neonatal intensive care unit

*further adjusted for presence of sexually transmitted infections (syphilis, gonorrhea, chlamydia, and/or genital herpes)

Among preterm infants, BV exposure was associated with a 24 % increased risk for admission to NICU after adjusting for gestational age, maternal age, and maternal race (Table 4). Assisted ventilation at birth, neonatal sepsis, infant and neonatal mortality were not associated with BV exposure.

**Table 4:** Associations between bacterial vaginosis and adverse outcomes among preterm infants (less than 37 weeks gestation), Washington State, 2003-2013, adjusted for gestational age, maternal age, and maternal race (*N* = 975)

	BV exposed		BV unexposed		RR	95 % CI	aRR	95 % CI
	*N* = 267		*N* = 708					
	*n*	*%*	*n*	*%*				
Assisted ventilation at birth	51	*19.1*	94	*13.3*	1.44	1.05–1.96	1.23	0.89–1.73
Admission to NICU at birth	124	*50.4*	246	*36.0*	1.40	1.20–1.64	1.24	1.04–1.46
Neonatal sepsis	47	*17.6*	82	*11.6*	1.52	1.09–2.11	1.31	0.91–1.89
Fetal Mortality	21	*7.9*	21	*3.0*	2.65	1.47–4.78	1.25	0.67–2.33
Infant mortality	17	*6.9*	21	*3.1*	2.26	1.21–4.22	1.10	0.66–1.84

*BV* bacterial vaginosis, *RR* risk ratio, *aRR* adjusted risk ratio, *CI* confidence interval, *NICU* neonatal intensive care unit

Among all infants, preterm birth was 55 % more likely to occur in women with BV (Table 5). Assisted ventilation at birth, admission to NICU, and neonatal sepsis were also associated with BV exposure in the whole cohort. There was an association between BV and 2nd trimester (20-28 weeks gestation) fetal mortality (aRR = 10.42, 95 % CI 3.55, 30.61).

**Table 5:** Associations between bacterial vaginosis and adverse outcomes among all infants, Washington State, 2003-2013, adjusted for maternal age and race (*N* = 12,340)

	BV exposed		BV unexposed		RR	95 % CI	aRR	95 % CI
	*N* = 2468		*N* = 9872					
	*n*	*%*	*n*	*%*				
Preterm birth	*267*	*10.8*	*708*	*7.2*	1.51	1.32–1.72	1.55	1.34–1.78
Assisted ventilation at birth	151	*6.1*	425	*4.3*	1.42	1.19–1.70	1.45	1.21–1.75
Admission to NICU at birth	213	*8.7*	519	*5.3*	1.65	1.42–1.92	1.73	1.47–2.03
Neonatal sepsis	95	*3.9*	209	*2.1*	1.82	1.43–2.31	1.86	1.44–2.41
Fetal Mortality	25	*1.0*	35	*0.4*	2.86	1.71–4.76	2.79*	1.54–5.05
* 2nd trimester*	16	*0.7*	7	*0.1*	12.22	4.29–34.79	10.42*	3.55–30.61
* 3rd trimester*	9	*0.4*	28	*0.3*	1.49	0.65–3.40	1.31*	0.55–3.11
Infant mortality	21	*0.9*	45	*0.5*	1.89	1.12–3.15	1.61	0.90–2.90

*BV* bacterial vaginosis, *RR* risk ratio, *aRR* adjusted risk ratio, *CI* confidence interval, *NICU* neonatal intensive care unit

*further adjusted for presence of a maternal comorbidity (hypertension, diabetes, and/or pre-eclampsia)

## Discussion

Consistent with prior research, we found a strong association between BV and risk for preterm birth [3, 5, 10, 11, 13]. More importantly, our primary study goal was to assess *term outcomes*. We hypothesized that BV would increase the risk for adverse neonatal outcomes, even among infants born full-term. We found an association between BV and an increased risk of neonatal assisted ventilation/respiratory distress, admission to NICU, and neonatal sepsis in the full cohort of term and preterm infants, similar to a prior study [6]. But, notably these findings persisted when analyses were restricted to only term deliveries.

Our data demonstrate an association between BV during pregnancy and poor neonatal outcomes at term that has only been hinted at previously [4]. We had hypothesized that poor neonatal outcomes could be due to ascending infection during pregnancy that might increase risks of chorioamnionitis or poor fetal growth, which in turn might increase the risk of poor neonatal outcomes. Meta-analyses previously showed a trend for increased risk of neonatal sepsis associated with BV in infants of all gestational ages, but results were non-significant and sample sizes limited with an inability to fully explore term outcomes [3, 5]. Data from a single study, with a small sample of 151 term BV exposures and 6 instances of infectious morbidity, suggests a modest association between infectious morbidity and BV exposure early in pregnancy among full-term infants; however, this association was not formally examined [4]. BV has been associated with chorioamnionitis in primarily preterm cohorts [14, 15], and chorioamnionitis is associated with neonatal sepsis in both term and preterm infants [16, 17]. Our study of 12,340 infants showed significant associations between BV and neonatal sepsis, both in the complete cohort and in the subset of full-term infants that included 2,198 BV-exposed mother/infant pairs. BV-exposed women delivering at term also had higher rates of meconium, which has been associated with in-utero bacteria [18] and worse neonatal outcomes [19]. However, when the presence of meconium and/or chorioamnionitis were included in our models, the risk for adverse outcomes persisted, suggesting that the association between BV and neonatal compromise is not due to meconium aspiration or the direct effects of chorioamnionitis. BV is associated with upper genital tract infection in both pregnant [20] and non-pregnant [21] women, thus sub-clinical infection could be one mechanism for the poor neonatal outcomes observed.

Others have not evaluated full-term births for the association of BV with SGA or LBW. Among full-term infants in our cohort, BV-exposed infants were more likely to be SGA, but not more likely than unexposed infants to have LBW. In our full cohort, BV-exposed infants were more likely to be SGA or LBW, confirming previous reports of this association [15, 22, 23]. In other studies among preterm infants, BV has been associated with LBW [1]. In a cohort of late preterm (>33 weeks) and term infants, SGA was associated with increased NICU admission, low Apgar scores and composite neonatal morbidity [24]. In our study, SGA infants were at greater risk of NICU admission when born preterm, and greater risk of neonatal sepsis when born full-term, but these increased risks were not statistically different from LGA and AGA infants.

The increased risk of NICU admissions and respiratory distress among full-term infants exposed to BV that we observed in our cohort has not been previously described, to our knowledge. In a small cohort of preterm infants, the proportions of infants with respiratory distress, requirement of intermittent positive pressure ventilation, admission to NICU, and NICU stays over two days was higher among BV exposed than among BV-unexposed pregnancies (uncontrolled analyses) [6]. We confirmed the association between BV and NICU admission in preterm infants, controlling for gestational age and several possible confounders, strengthening the likelihood that this association is biologically true.

We did not find an increased risk for term perinatal mortality associated with BV and, to our knowledge, this has not been studied previously. BV has not been associated with perinatal mortality in previous meta-analyses of primarily preterm cohorts [3]. Perinatal mortality was not associated with overall fetal or infant mortality in our full cohort. However, when we evaluated mortality by trimester, the risk of 2nd trimester fetal mortality was increased, supporting meta-analyses that reported a 6-fold increased risk for loss between 14-24 weeks in women diagnosed with BV during early pregnancy [3].

Our study has strengths and limitations. It was large, population-based, and rich in covariates, with little missing data. While birth certificate sensitivity is poor for some variables [8, 25], we increased ascertainment of exposures and outcomes by using both birth certificates and linked hospital records. Since routine screening for BV is not standard of care, cases identified were likely symptomatic, which may limit this study’s findings to cases of symptomatic BV. While asymptomatic cases may be under-diagnosed, including asymptomatic BV-exposed mothers among the unexposed would bias results towards the null. We did not have data on BV treatment in exposed women or the trimester that BV was diagnosed. If BV treatment tempers the increased risk of the poor neonatal outcomes associated with BV, associations may be even greater than reported here. Furthermore, associations may differ according to the timing of BV treatment. Further studies are needed to address these questions. Additionally, 68 % of the women were White, limiting the generalizability of our findings. A large proportion of women were nulliparous. But as all data are de-identified, mothers who gave birth more than once during the study period may have been included multiple times, potentially introducing a clustering bias we are unable to address. Lastly, our study shows an association, but cannot confirm a causal relationship between BV and poor outcomes among term deliveries.

## Conclusions

BV during pregnancy appears to increase the risk for adverse neonatal outcomes through mechanisms independent of BV’s effect on timing of delivery. These findings may have significant clinical implications if confirmed by other studies, but several questions remain, including the importance of timing of the exposure and the mechanism for these associations. While treatment of BV during pregnancy has not succeeded in reducing preterm birth [26, 27], treatments targeted specifically at improving term neonatal outcomes have not been explored as thoroughly. The Maternal Fetal Medicine Unit's treatment trial for asymptomatic BV between 16 and 24 weeks did not show a difference in birthweight, NICU admission, neonatal sepsis, or neonatal death, though data were only shown for the first outcome. The sample size of the trial was only 1,953, thus it may not have had adequate power to detect differences in these uncommon outcomes [27]. A Cochrane review [28] of seven randomized clinical trials on antibiotic prophylaxis during pregnancy for women at high risk of preterm birth had inadequate power to study the effect of prophylaxis on neonatal sepsis, did not restrict analyses to women with BV, did not report on NICU admission, and did not focus on term deliveries. There are very few prior studies directly relevant to our findings - an increased risk of neonatal assisted ventilation/respiratory distress, admission to NICU, and neonatal sepsis among BV exposed infants born at term. Our findings suggest that any study evaluating the vaginal microbiota in pregnancy should include neonatal outcomes as an endpoint, and expand our understanding of BV associated outcomes in preterm infants to term deliveries.

## Abbreviations

AGA: Average for gestational age
aRR: Adjusted relative risk
BERD: Birth Events Record Database
BV: Bacterial vaginosis
CHARS: Comprehensive Hospital Abstract Reporting System
ICD: International Classification of Disease
LBW: Low birth weight
LGA: Large for gestational age
NICU: Neonatal intensive care unit
RR: Relative risk
SGA: Small for gestational age
STI: Sexually transmitted infection
